# Robotic Bilateral Ileal Interposition: The Total Intracorporeal Approach

**DOI:** 10.1590/S1677-5538.IBJU.2025.0359

**Published:** 2025-07-30

**Authors:** Chi-Hang Yee, Ivan Ching-Ho Ko, Ho-Man Tam, Ho-Fai Wong, Ka-Lun LO, Wilson Hoi-Chak Chan, Alex Qinyang Liu, Jeremy Yuen-Chun Teoh, Peter Ka-Fung Chiu, Chi-Fai Ng

**Affiliations:** 1 Prince of Wales Hospital Department of Surgery Shatin Hong Kong Department of Surgery, Prince of Wales Hospital, Shatin, Hong Kong

## Abstract

**Purpose:**

While grafting procedures can be used to manage upper ureteric strictures (1), ileal interposition still has its role in the more complex cases with long or bilateral ureteric strictures. Ketamine-associated uropathy can mimic retroperitoneal fibrosis and give rise to long bilateral ureteric strictures (2, 3). The objective of the video is to present the total intracorporeal technique for bilateral ileal interposition with a U-configuration of small bowel loop.

**Patient and methods:**

A 34-year-old lady with bilateral long ureteric stricture secondary to ketamine-associated uropathy was managed with robotic reconstruction. The surgical access to retroperitoneum and exposure of bilateral ureters took reference from the single-docking robotic retroperitoneal lymph node dissection technique (4). Da Vinic Xi robotic system was used. Retroperitoneal space was accessed after incision was started behind the caecum, lifting up the root of small bowel mesentery. Bilateral ureters were exposed and the segment proximal to the stricture was identified. In the literature, different techniques of small bowel isolation have been described (5, 6). In this case, a loop of small bowel in U-configuration was prepared and the end of both limbs was anastomosed to the ureters in a side-to-end manner. The apex of the bowel loop was anastomosed to the bladder, completing the procedure.

**Results:**

The procedure took 507 minutes. Hospital stay was 8 days. Unobstructed drainage was confirmed by DTPA scan at 3 months and 15 months. UUnremarkable drainage was noted on IVU at 6 months and on CT scan at 18 months. Renal function was static at 3 years with creatinine clearance 56mL/min. The patient was symptom free at post-operative 3 years.

**Conclusions:**

Retro-caecal exposure of the retroperitoneal plane allows a convenient access to ureters. Bilateral ileal interposition can be safely performed in a minimally invasive manner with robotic technique.

Available at: http://www.intbrazjurol.com.br/video-section/20250359_Yee_et_al

**Video 1:** 

## References

[1] Chao BW, Raver M, Lin JS, Zhao K, Lee M, Gelman S (2025). Robotic Buccal Mucosa Graft Ureteroplasty: A Decade of Experience From a Multi-institutional Cohort. Urology.

[2] Yee CH, Teoh JY, Lai PT, Leung VY, Chu WC, Lee WM (2017). The Risk of Upper Urinary Tract Involvement in Patients With Ketamine-Associated Uropathy. Int Neurourol J.

[3] Yee CH, Lai PT, Lee WM, Tam YH, Ng CF (2015). Clinical Outcome of a Prospective Case Series of Patients With Ketamine Cystitis Who Underwent Standardized Treatment Protocol. Urology.

[4] Mittakanti HR, Porter JR (2020). Robot-assisted laparoscopic retroperitoneal lymph node dissection: a minimally invasive surgical approach for testicular cancer. Transl Androl Urol.

[5] Faria EF, Maciel CVM, Melo PA, Tobias-Machado M, Dias RM, Dos Reis RB (2024). Mesentery-Sparing Technique: a New Intracorporeal Approach for Urinary Diversion in Robot-Assisted Radical Cystectomy. Int Braz J Urol.

[6] Gaya JM, Uleri A, Sanz I, Basile G, Verri P, Hernandez P (2023). Robot-assisted radical cystectomy and ileal conduit with Hugo™ RAS system: feasibility, setting and perioperative outcomes. Int Braz J Urol.

